# A Nanometer Water Pump Induced by the Brownian and Non-Brownian Motion of a Graphene Sheet on a Membrane Surface

**DOI:** 10.1186/s11671-018-2732-x

**Published:** 2018-10-01

**Authors:** Chang Fang, Fujing Lv, Jiaye Su

**Affiliations:** Department of Applied Physics, Nanjing University of Science and Technology, Nanjing, 210094 Jiangsu China

**Keywords:** Water, Transport, Carbon nanotube, Molecular dynamics

## Abstract

Energy-saving water pump and efficient semipermeable membranes are the cores of reverse osmosis technology. Applying nanotechnology to improve the performance is a fashion in recent years. Based on the competitive effect of water’s spontaneous infiltration of two sides of a carbon nanotube, we design a water pump that makes use of the natural permeability by weakening one side’s competitiveness based on a small graphite sheet laying on the membrane. According to molecular dynamic simulations, continues net flux is observed. The motion mode of the sheet is the key for the performance. For the pure Brownian motion without any dynamical load, we find two water molecules per nanosecond flux, while the flux induced by the unidirectional motion can be several times enhanced, depending on the external force. The Brownian motion is similar to the physical mechanism of osmotic pressure, and the unidirectional motion shows great performance that has huge applications for reverse osmosis. Our work creatively proposes a new strategy to pump water molecules crossing though a nanochannel, inspiring for nanofluidic device designers.

## Background

Seawater desalination is a highlighter direction to solve the global water shortage for it can offer endless clean water in theory. However, the current technology is not perfect. There are two different methods for seawater desalination. The first is distillation, obtaining pure water by heating seawater and then cooling the vapors. Phase changing can fully remove impurities but with energy intensive and expensive. The other one is reverse osmosis (RO), driving seawater through a semipermeable membrane that is permeable to water but impermeable to ions. Benefits from the improving of semipermeable membranes and pressure water pumps, RO is mature and widely used [1]. However, RO is still energy intensive [2–4]. This is because RO system needs keeping a high pressure drop to make up permeable pressure and drive seawater through semipermeable membranes. Many scientists believe that “in order for desalination to live up to the water challenges of the 21th century a step-change is needed in RO membrane technology” [5]. They propose that carbon nanotubes (CNTs) are an ideal water channel with the advantages like selectivity, high efficiency, and low energy cost [6] and have great potential for applications as nanofluidic channels [7–10]. However, only improving the RO membrane property is helpful for effectiveness of RO but useless for save energy as current RO desalination has already near the thermodynamic limitation [4]. More efficient driving methods are needed as alternatives to high-pressure pump [11].

For a CNT channel connecting two water reservoirs, water molecules always can enter the channel spontaneously due to the Brownian motion. However, the infiltration effects of two sides of CNT channel offset each other as no net water flux exists. As the net flux is regarded by the competition result of Brownian motion of two sides of CNT channel, enhancing or weakening one side competitiveness should be an effective method for pumping water. In previous work, pressure drop [12, 13], temperature difference [14, 15], and electric field [16] are common strategies to enhancing the competitiveness on one side to create net water flux. Nonetheless, weakening the competitiveness seems to be a better choice as we make use of the natural permeability.

Actually, controlling the nanofluidic transport is relevant to widespread applications, ranging from energy storages to biosensors [17–23], which is still a challenge. Herein, we design a new water pump with a small graphite sheet on one membrane side with the goal of breaking the balance of Brownian motion of the two reservoirs, resembling a symmetric breaking system. The sheet has two motion modes: thermal motion and unidirectional motion, corresponding to Brownian and non-Brownian motions, respectively. By simulation calculations, weakening the top side competitiveness is achieved and a down-to-top water flux is induced. Besides, for the Brownian motion, the amount of water flux is nearly two per nanosecond that is close to aquaporin [24, 25], suggesting possible applications in biological membranes. The small sheet drives water from down-to-top side through the CNT, which can be analogy to the physical mechanism of osmotic pressure. Furthermore, in the unidirectional motion, the flux amount can be significantly enhanced by several times, depending on the sheet moving speed or external force. As the technology enters molecular scale operation, such as the manipulation of surface nanoparticles by optical tweezers [26] and atomic force microscopy [27], our work displays a probability of tuning the water permeation symmetry, which opens a new method for water pump.

## Model and Simulation Method

**Fig. 1:**
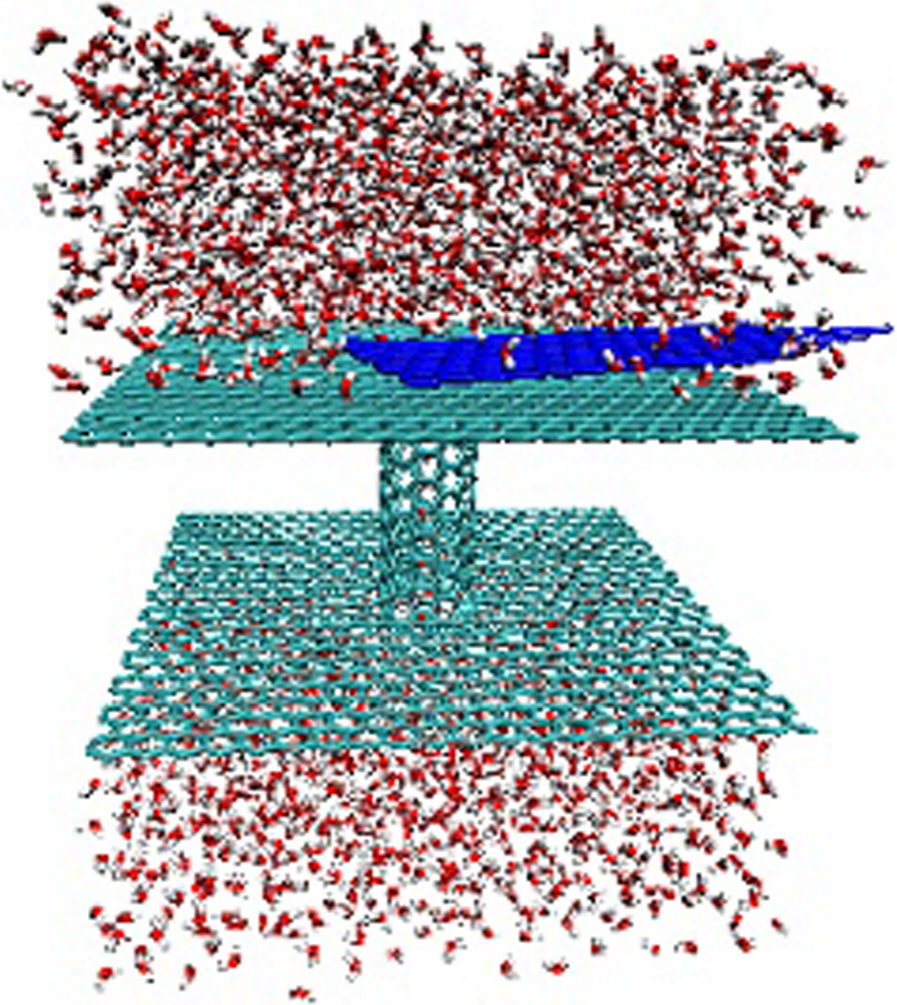
Snapshot of the simulation system. A CNT with length of 2.56 nm and diameter of 0.81 nm, connecting two water reservoirs, separated by two graphite sheets (sage green, 5.1 × 5.1 nm^2^). A small graphite sheet (blue) placed on the big one closely. The system was embedded in a periodic water box with 3328 water molecules, representing a nanometer water pump

A snapshot of the simulation system is shown in Fig. 1. We use a (6, 6) CNT (length of 2.56 nm and diameter of 0.81 nm) and two parallel graphite sheets (5.1 × 5.1 nm^2^) to compose a permeable membrane. In such a narrow channel, water molecules exhibit a single-file arrangement [6]. A small graphite sheet made up of 272 carbon atoms is placed on the membrane closely. The strong carbon-carbon interaction leads to the adsorption of a small sheet on the membrane. In fact, during the process of our simulations, the mean distance of the sheet and membrane is about 0.34 nm. In the Brownian motion, we set the temperature of the small graphite sheet ranging from 100 to 500 K. It will oscillate on the membrane near the CNT entrance, colliding with the nearby water molecules. Three thousand three hundred twenty-eight water molecules fill the channel and two reservoirs. The temperature of water is fixed as 300 K. For the unidirectional motion mode, we apply an additional acceleration on each carbon atom of the small sheet to achieve the additional force, where 0.1 nm/ps^2^ is corresponding to 2 pN. The additional force is along *x* direction. Water flux is induced by the asymmetry of the system. Due to the periodic boundary condition in all the three dimensions, the sheet will be continuously passing through the vicinity of CNT entrance, and induces stable water flow and flux.

During our simulations, the system was in a constant volume and temperature with periodic box, and water molecules were classical TIP3P models [28]. Carbon atoms were uncharged Lennard-Jones (LJ) particles with parameters of *σ*_cc_ = 0.34 nm, *ε*_cc_ = 0.3612 kJ/mol; *σ*_co_ = 0.3275 nm, *ε*_co_ = 0.4802 kJ/mol [6]. PME method was employed to deal with the long-range electrostatic interactions [29]. The simulations run 125 ns on the software of Gromacs 4.6.5 [30] with a time-step of 2 fs (data collected every 1 ps), and the last 120 ns was gathered. Two independent simulations were done to reduce error.

## Results and Discussion

### The Brownian Motion of a Graphite Sheet

**Fig. 2:**
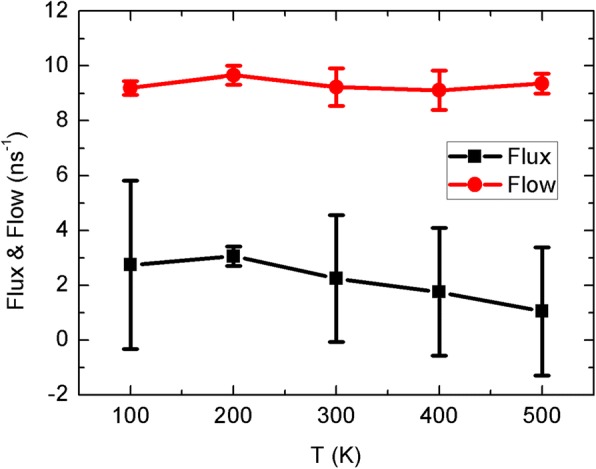
The water flux and flow as a function of the sheet temperature. Error bars are shown for two data points

At first, we study the Brownian motion mode of the sheet at different temperatures. To measure the ability of inducing water flux through CNTs, following the previous work [31, 32], we define the upflux and downflux as the amount of water molecules conducting through the tube along the + *z* and − *z* direction, respectively. Flow = upflux + downflux, flux = upflux − downflux, and unidirectional transport efficiency *η* can be calculated by *η* = flux/flow. The water flow and flux as a function of the sheet temperature are shown in Fig. 2. In our original hypothesis, the hot sheet heats the water around and then creates temperature difference along the CNT to drive water through the channel. However, the water flux in the simulations is down-to-top, which is opposed to what we expected. Besides, the water flux is insensitive to the sheet temperature. Moreover, the temperature fluctuation of a small sheet is within the scope of 10 K during our simulation process. Actually, due to the temperature control of NVT simulations, the heat exchange between the sheet and surrounding solution is weak and can be ignored. As Fig. 2 exhibits, we can always obtain continuous net flux at about two water molecules per nanosecond no matter what the sheet temperature is, which is close to the experimental value of 1.8 in aquaporin channels [24, 25], suggesting potential applications in biological systems. Meanwhile, the total water flow is almost independent of the sheet temperature and should be similar to the case without sheet.

The bias water transport by the Brownian motion of a nanosheet is resembling the osmotic process. From the perspective of molecular dynamics, the net water flux should be caused by the competition of Brownian motion of water molecules near the two entrances of the CNT channel. The small sheet influences the velocity of water molecules by frequent collisions and then changes the competitiveness. Interestingly, the sheet is placed on the top side but induces the down-to-up water flux, suggesting the effect of the sheet is weakening the competitiveness. However, the Brownian motion of the sheet is irregular and the net flux is insensitive to the temperature with large fluctuation. Hence, we will further discuss the unidirectional motion mode of the sheet in the next part, and more interesting phenomena are discovered.

**Fig. 3:**
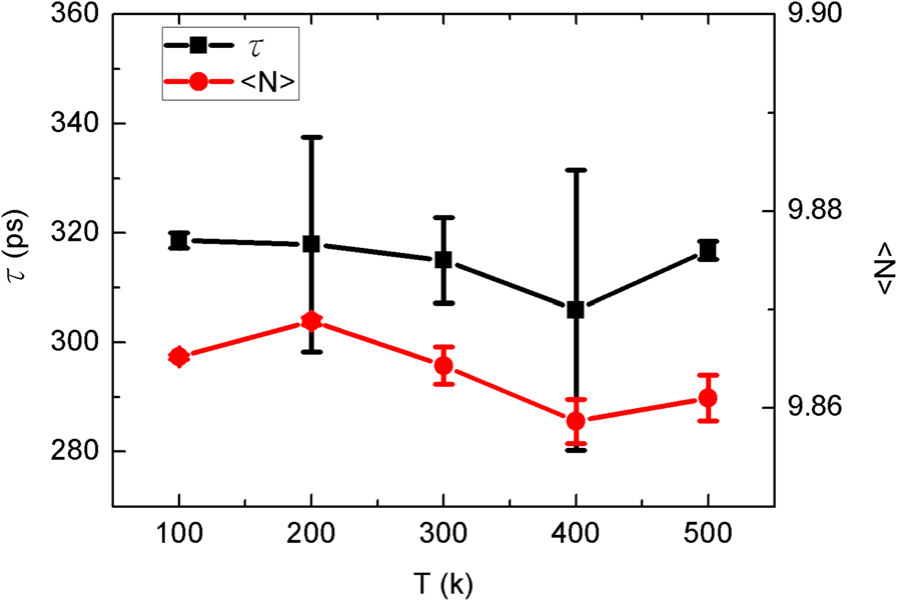
The water translocation time *τ* and occupancy <*N*> as a function of the sheet temperature

Then, we gather the water translocation time and occupancy as seen in Fig. 3. Here, the translocation time is the average transit time for water molecules through the CNT channel. Similar to the water flow, the translocation time fluctuates with the sheet temperature. In fact, the translocation time should be corresponding to the water flow, as the more quickly water molecules pass through the channel the larger water flow should be. Nonetheless, such an anti-relation is covered by the thermodynamics fluctuation herein. In theory, the occupancy is determined by the structure of CNT channel. As the single-file water chain is maintained, there are always nearly ten water molecules inside the CNT channel with slight fluctuations. Hence, thermodynamic fluctuations are inevitable but not remarkable.

**Fig. 4:**
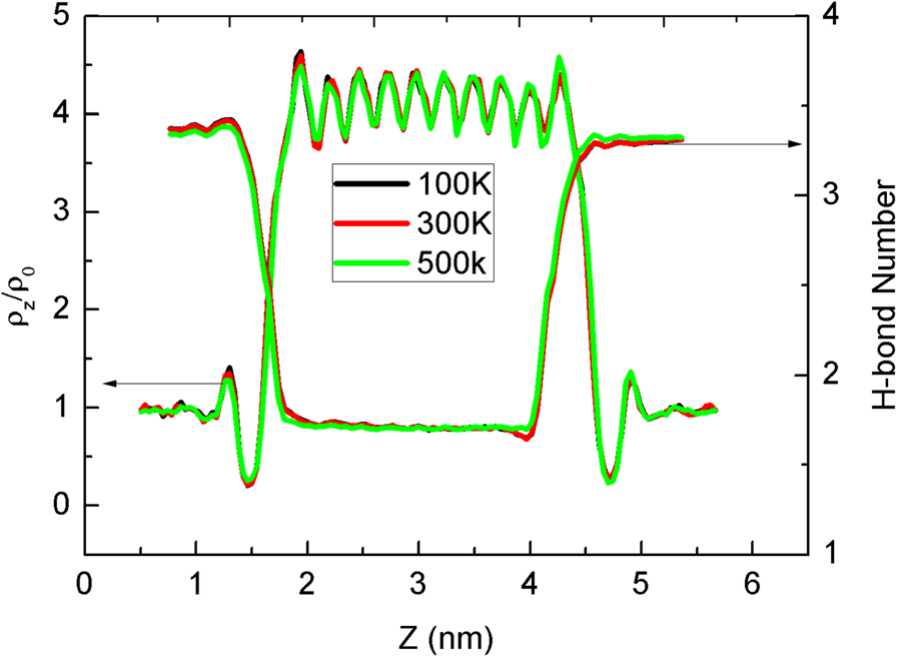
Density and hydrogen bond number distribution along *z* axis and different line colors are for different sheet temperatures. Here, *ρ*_0_ is 1.0 g/cm^3^ of the bulk water density

As thermal dynamics properties of water molecules inside narrow CNT is another important issue we concern, density distributions and hydrogen bond (H-bond) number are counted as a function of *z* position displayed in Fig. 4. Here, the two water molecules form a H-bond when their oxygen distance is less than 0.35 nm and the angle between O–H bond and O–O is less than 30°. The 2–4 nm part of *z* position corresponds to the CNT channel, where the density and H-bond number behavior is different from bulk areas. The density in the CNT is almost four times as much as in bulk, implying the potential of mass storage. The wave-like pattern of density with ten peaks is in line with the occupancy in Fig. 3, owing to the unique CNT structure. The change of H-bond number also displays the process of a water molecule entering into the CNT to form a single-file chain with reduced H-bonds.

**Fig. 5:**
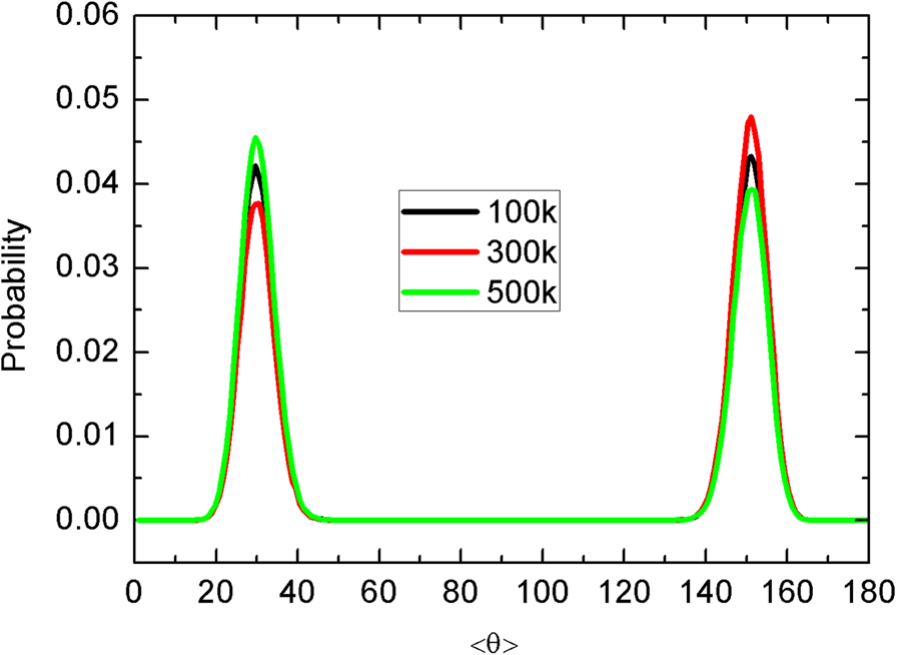
Probability distribution of the average dipole orientation of water molecules inside the CNT and different sheet temperatures are marked by line colors

Water molecules inside the CNT with unique orientations have been revealed early [16]. Here, we calculate the probability distribution of the water-dipole orientations as shown in Fig. 5. To reduce the error, we average the data from the two independent simulations. <θ> is the averaged angle between water dipole and the *z* axis, and there are almost two states (20°–40° and 140°–160°) for the water orientations. The pattern is almost symmetric with respect to <θ> = 90°, indicating the unique dipole orientation. As a whole, the transport of water is insensitive to the sheet temperature. This is because the Brownian motion of the sheet is always on the graphene membrane due to the strong sheet-membrane hydrophobic interaction, and the impact of the sheet is very limited. In the following, we will further discuss the unidirectional motion mode of the sheet, where the water transport can be affected more significantly.

### The Unidirectional Motion of a Graphite Sheet

**Fig. 6:**
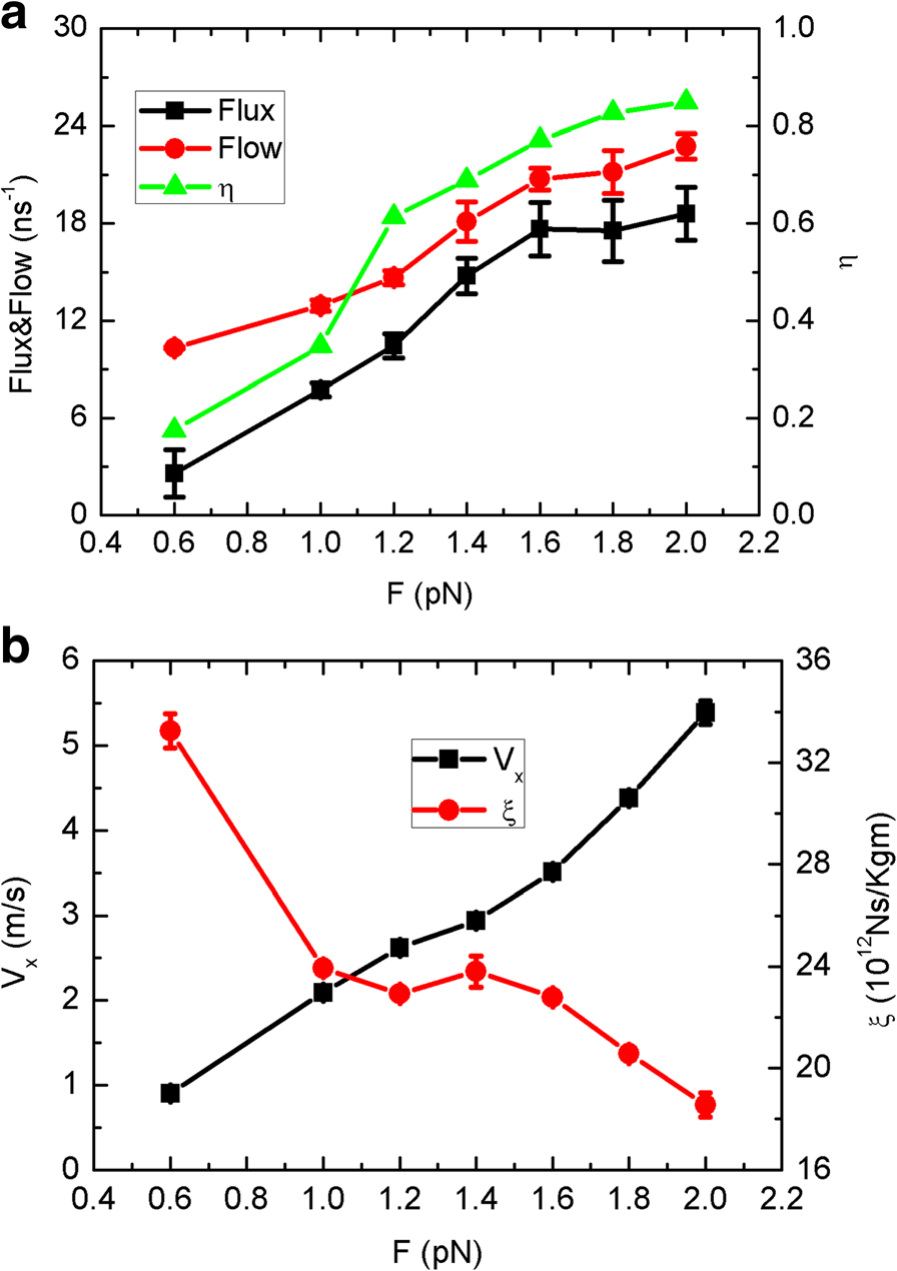
**a** The water flux, flow, and unidirectional efficiency *η* and **b** the sheet velocity *V*_*x*_ and friction coefficient *ξ* as a function of the driving force *F*

As the motion of the sheet should be important for the performance, we further investigate a typical non-Brownian motion mode, i.e., the unidirectional motion. The sheet is driven by an additional force and moves on the graphene membrane with stable velocity. Interestingly, the water flow, flux, and unidirectional transport efficiency *η* increase quickly with the increase of force as seen in Fig. 6a. Then, to describe the dynamics of the sheet, we introduce the one-dimensional Langevin equation:

$$ m\frac{d^2x}{\mathrm{d}{\mathrm{t}}^2}=F- m\xi \frac{\mathrm{d}\mathrm{x}}{\mathrm{d}\mathrm{t}}+R(t) $$

where, *m* is the sheet mass, *F* is the driving force, *R* (*t*) is the force caused by the random collisions of water molecules, and *ξ* is the friction coefficient. The random collisions is complicated, and here, we just count *R*(*t*) as the mutually offset collisions and <*R*(*t*)> = 0. In the steady state, the sheet keeps a uniform speed, and the friction force is equal to the driving force. Thus,

$$ F= m\xi \frac{\mathrm{dx}}{\mathrm{dt}}= m\xi v $$

We display the velocity (from the MD trajectory) and the friction coefficient *ξ* as a function of the driving force in Fig. 6b. The velocity increases almost linearly with the driving force, corresponding to the behaviors of flux and flow, while the friction coefficient decreases as a whole. Thus, the water flow and flux should be directly related to the sheet velocity. In the view of molecular dynamics, as the competitive effect exists, the sheet drags the surrounding water molecules and weakens the competitiveness of top side. The quicker the sheet moves, the weaker the competitiveness should be. As the force exceeds 1.6 pN, the flux tends to be gentle, close to 16 per nanosecond that is nearly 8 times than the Brownian mode. Obviously, this unidirectional motion is more efficiency than the random Brownian motion. Therefore, the artificially controlled sheet is another alternative strategy for reverse osmosis, where the sheet could be manipulated by some advanced experimental technologies such as optical tweezers [26] and atomic force microscopy [27].

**Fig. 7:**
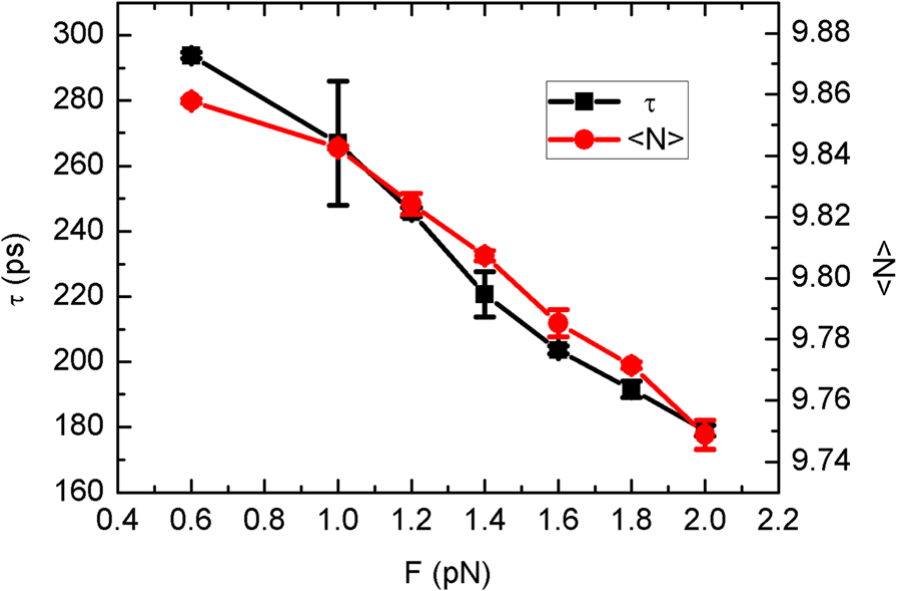
The translocation time *τ* and occupancy <*N*> as a function of the driving force

Remarkably, the increasing of the sheet velocity and driving force lead to the weakening of the competition of top side much more than the Brownian mode. In an effort to further elucidate how the water traveling is affected, we display the average translocation time and occupancy as a function of the driving force in Fig. 7. Both of them show nearly linear relations with the driving force, differing from the results of Fig. 3. The decay of translocation time is corresponding to the increasing behavior of water flow in Fig. 6a, which should be caused by the drag of the sheet. From another point of view, when the sheet drags surrounding water molecules, thermal competitiveness of top side should be reduced, facilitating the down-to-top water permeation though the CNT channel.

**Fig. 8:**
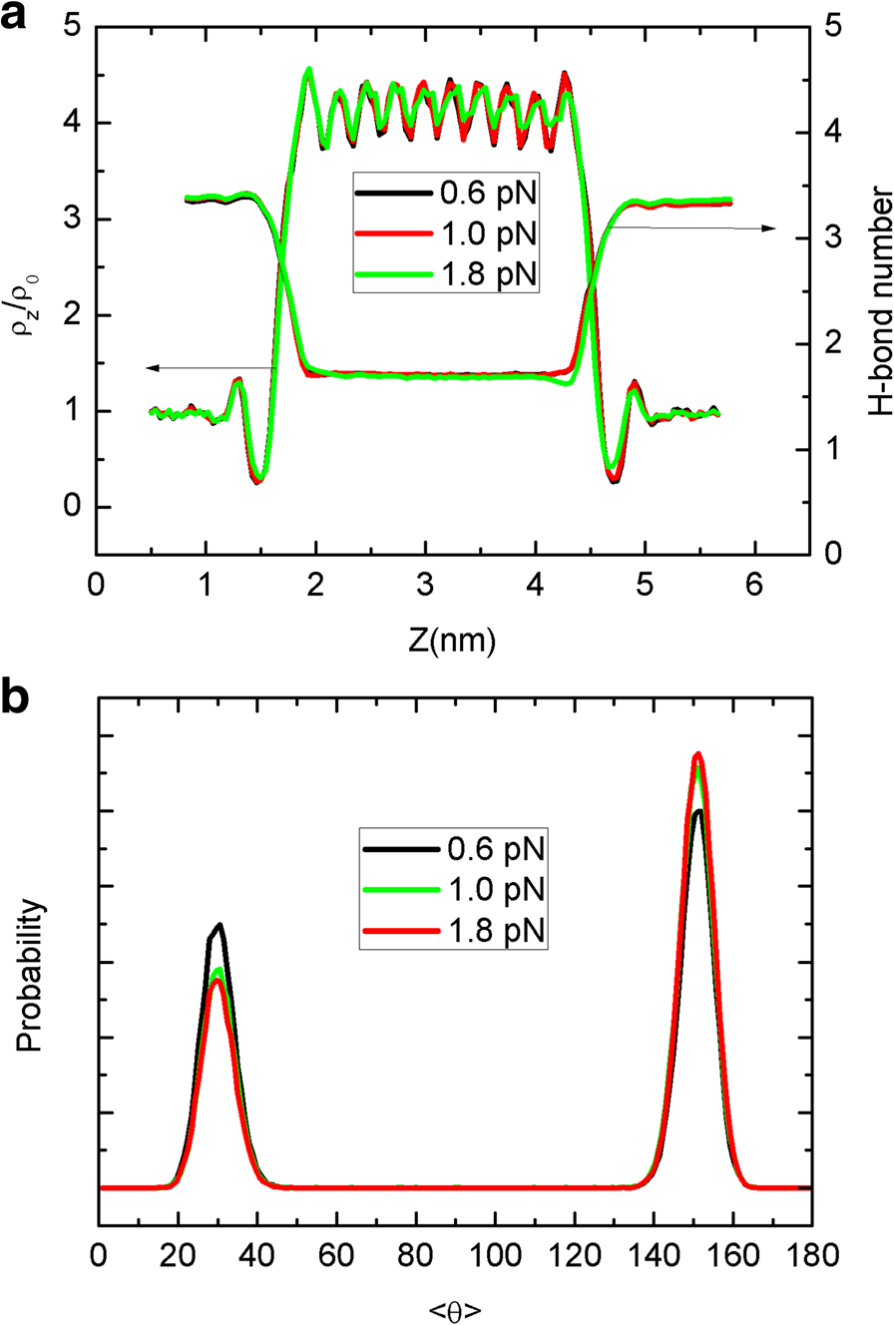
**a** The axial water density profiles and hydrogen bond number as a function of *z* position along the CNT for different force. **b** Probability distribution of the average dipole orientation of water molecules inside the CNT for different force

We further present the water density profiles, H-bond, and water dipole distributions in Fig. 8. As can be seen in Fig. 8a, the density profiles and H-bond are only slightly affected by the sheet motion. For example, under the large force of 1.8 pN, the wave-like density peaks become reduced and the H-bond distribution becomes slightly asymmetric. Similar change can be found for the water dipole orientation in Fig. 8b. Under the equilibrium condition, e.g., for the Brownian motion above, the two orientation events happen with similar probability, leading to the similar peak height, as seen in Fig. 5. However, as we have discussed, the unidirectional motion of the sheet should have more influence on the water chain than the Brownian motion. This is because the flowing sheet will drag the surrounding water to move due to the sheet-water Lennard-Jones interaction and thus affect the motion or orientation of water near the CNT entrance. Therefore, the dipole orientations in Fig. 8b become asymmetric. Although the dynamics and thermodynamics of confined water can be disturbed more deeply for the unidirectional motion, due to the preservation of single-file water chain, such a disturbance is still very limited especially for the thermodynamics and the key features of density, H-bond, and dipole are close to the case of Brownian motion. Consequently, different motion mode of the sheet can have a higher impact on the water dynamics rather than thermodynamics.

### Additional Discussion

**Fig. 9:**
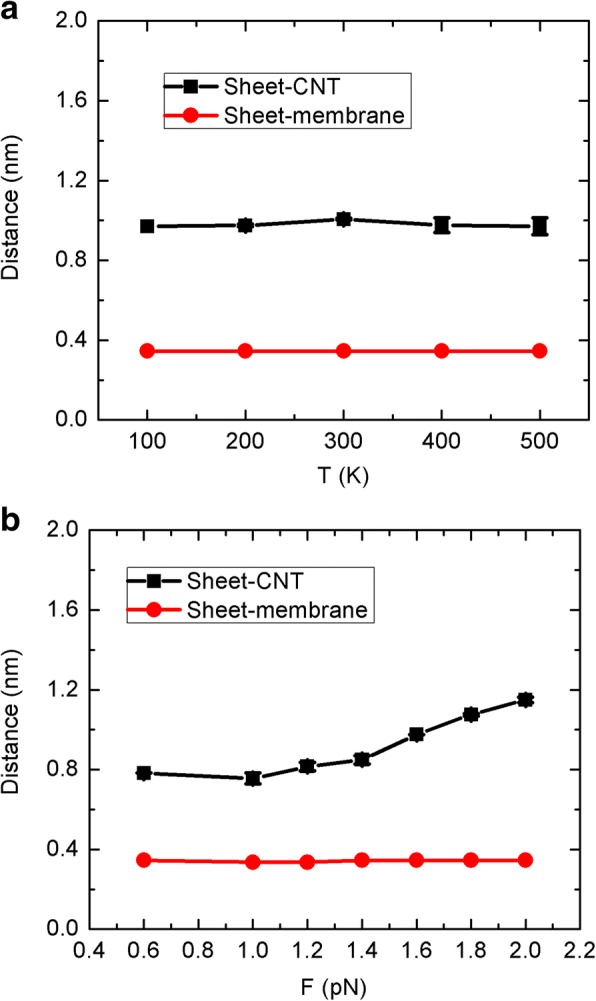
The mean distance of sheet-membrane and sheet-CNT for different simulation conditions: **a** Brownian motion and **b** unidirectional motion

It is believed that the initial distance between the graphene sheet and CNT entrance should have trivial effect on the water flow and flux through the CNT. We actually randomly put the sheet on the top graphene membrane, where the sheet is directly adsorbed on the surface without any water in-between, as seen in Fig. 1 above. In this way, the sheet will always move on the surface because of strong sheet-membrane hydrophobic interaction, providing an asymmetric nanofluidic system. As shown in Fig. 9, we calculated the mean distance of sheet-membrane and sheet-CNT for both of the Brownian and unidirectional motions. It is striking that the mean distance of sheet-membrane is fixed at 0.34 nm for both cases, strictly corresponding to the carbon-carbon Lennard-Jones potential diameter. Thus, the sheet will be always adsorbed on the membrane surface. For the Brownian motion in Fig. 9a, the distance of sheet-CNT is also a constant that is independent of the sheet temperature. This is clearly due to the sheet-CNT hydrophobic interaction that leads to the sheet encircling with respect to the CNT. We should also note that in our simulation setup, the CNT entrance exceeds the location of graphene membrane 0.2 nm, and this can well prevent the entrance being blocked by the sheet. It is believed that if the sheet is not initially put onto the membrane, it can move in the reservoir randomly and should have some probability of blocking the CNT entrance. Furthermore, for the unidirectional motion in Fig. 9b, the distance of sheet-CNT exhibits increasing behaviors with the increase of force, corresponding to the flow and flux behaviors. Under small force, the sheet can be actually trapped near the CNT for a while, while the larger force can faster the passing of sheet, leading to a larger distance. Overly, the initial distance of sheet-CNT should not have appreciable effect on the water flow and flux, while the sheet-membrane could have. However, if the sheet is initially in bulk water instead of on the membrane, the system should become symmetric that differs from our initial goal, and the bias transport phenomenon should disappear.

**Fig. 10:**
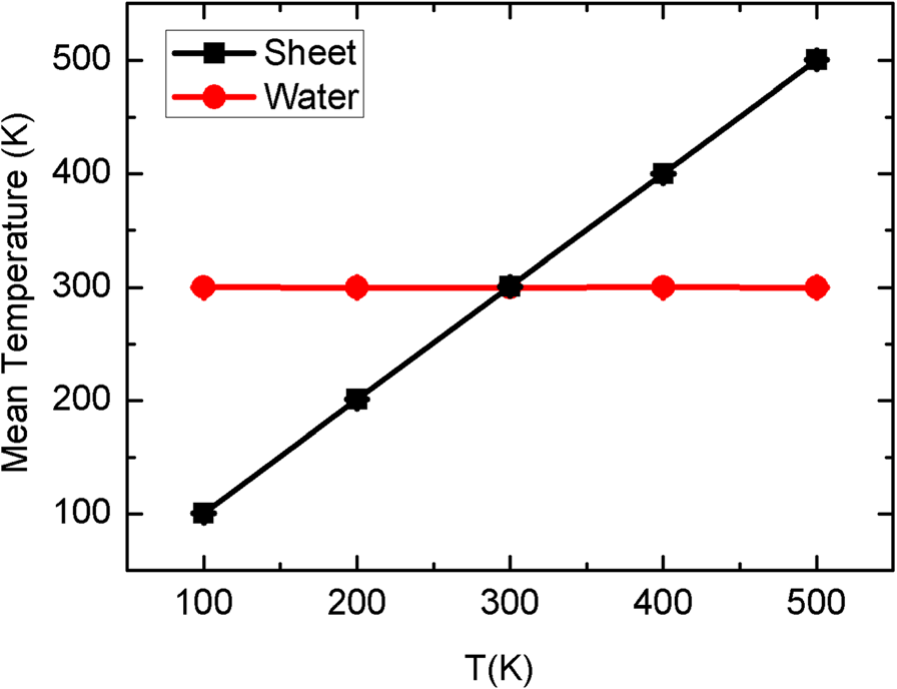
The mean temperatures of sheet and water as a function of the target sheet temperature

For the Brownian motion, the mean temperatures of water and sheet during the process of simulation are shown in Fig. 10 as a function of the target sheet temperature. We can see that the mean temperature of sheet can be strictly controlled at its target values, and similarly, the mean value of water is also maintained at *T* = 300 K. In fact, we used Nose-Hoover method to control the temperatures of both sheet and water. Generally, in the NVT (or NPT) ensembles of MD simulations, the heat exchange between different molecules cannot happen because of the thermostat. However, the intermolecular collisions between the sheet and surrounding water should exit, even if they are ultimately tuned by the thermostat. The collisions from the moving sheet can affect the instant velocity or its direction of surrounding water molecules and thus ultimately change the probability of water entering into the CNT. Nonetheless, it is still very difficult to capture such an instant influence of sheet on water, since it should happen in a very short time, possibly less than the data collection time of 1 ps. Thus, we may hypothesize that the sheet vibration can affect the thermal fluctuation of surrounding water and weaken the competitiveness of the top reservoir, leading to the bias transport phenomenon.

## Conclusions

In summary, we proposed a new strategy for water pump by molecule dynamic simulations and achieved a considerable net water flux based on the spontaneous water permeability. Water molecules enter in CNT channel initiatively due to Brownian motion, while two sides of CNT compete with each other and offset. In our research, a small sheet moving on the membrane weakens the competitiveness of one side and induces continuous net flux. During the simulations, we find the motion mode of the sheet is the key to the performance. The pure Brownian motion induces a small stable net water flux around 2 ns^−1^ that is independent of the sheet temperature, while the unidirectional motion can create significantly higher flux, depending on the driving force on the sheet. Furthermore, with the increase of the driving force, the water translocation time reduces linearly, corresponding to the water flow or flux behavior. Overly, the unidirectional motion has a higher impact on the water dynamics and thermodynamics. Consequently, we creatively presented making use of the nature water’s permeability, achieved by a small graphite sheet laid on the membrane, which would be helpful for the RO technology.

## Abbreviations

CNT: Carbon nanotube
MD: Molecular dynamics
RO: Reverse osmosis
